# Key stakeholder perspectives on HA Go implementation: a Consolidated Framework for Implementation Research–informed study of a mobile health navigation tool

**DOI:** 10.1093/jamiaopen/ooag133

**Published:** 2026-09-10

**Authors:** Claire Chenwen Zhong, Man Kin Yim, Mingtao Chen, Chung Yi Lo, Xiaoshu Zhang, Junjie Huang, Martin C S Wong

**Affiliations:** The Jockey Club School of Public Health and Primary Care, Faculty of Medicine, The Chinese University of Hong Kong, Shatin, Hong Kong SAR 999077, China; Centre for Health Education and Health Promotion, Faculty of Medicine, The Chinese University of Hong Kong, Shatin, Hong Kong SAR 999077, China; The Jockey Club School of Public Health and Primary Care, Faculty of Medicine, The Chinese University of Hong Kong, Shatin, Hong Kong SAR 999077, China; The Jockey Club School of Public Health and Primary Care, Faculty of Medicine, The Chinese University of Hong Kong, Shatin, Hong Kong SAR 999077, China; The Jockey Club School of Public Health and Primary Care, Faculty of Medicine, The Chinese University of Hong Kong, Shatin, Hong Kong SAR 999077, China; The Jockey Club School of Public Health and Primary Care, Faculty of Medicine, The Chinese University of Hong Kong, Shatin, Hong Kong SAR 999077, China; The Jockey Club School of Public Health and Primary Care, Faculty of Medicine, The Chinese University of Hong Kong, Shatin, Hong Kong SAR 999077, China; Centre for Health Education and Health Promotion, Faculty of Medicine, The Chinese University of Hong Kong, Shatin, Hong Kong SAR 999077, China; The Jockey Club School of Public Health and Primary Care, Faculty of Medicine, The Chinese University of Hong Kong, Shatin, Hong Kong SAR 999077, China; Centre for Health Education and Health Promotion, Faculty of Medicine, The Chinese University of Hong Kong, Shatin, Hong Kong SAR 999077, China

**Keywords:** digital health; implementation science; Consolidated Framework for Implementation Research; health care navigation, patient education

## Abstract

**Objectives:**

The Hospital Authority in Hong Kong developed an application, known as ‘HA Go’, to enhance patients’ healthcare journeys and optimize the efficiency of healthcare resource allocation. This study aimed to explore the perceptions of HA Go among the key stakeholders and identify barriers and facilitators to its adoption.

**Materials and Methods:**

This qualitative study evaluated the perceptions of six groups of key stakeholders associated with HA Go in Hong Kong including (1) users; (2) non-users; (3) caregivers; (4) physicians in the public healthcare sector; (5) physicians in the private healthcare sector; and (6) policymakers related to HA Go development. The Consolidated Framework for Implementation Research (CFIR) was adopted to guide the interview and data analysis in this study.

**Results:**

A total of 33 participants were included in this study. Among users, non-users, and caregivers, the key benefits of HA Go and facilitators included appointment management, simplification of the healthcare process, and accessible HA Go-related information and support. Among physicians and policymakers, the key benefits and facilitators included improved appointment coordination, smoother healthcare processes, and support for medication management. Across stakeholder groups, barriers included limited familiarity with digital devices, interface complexity, privacy concerns, and insufficient promotion or practical support.

**Discussion:**

Stakeholders generally perceived HA Go as useful for improving healthcare navigation and resource efficiency, while highlighting the need to address usability barriers and strengthen support for diverse user groups.

**Conclusions:**

This study highlighted the potential role of HA Go in enhancing patients’ healthcare journeys and optimizing healthcare resource allocation, and identified the enablers, barriers, and opportunities for improvement. Targeted improvements in user interface design, platform integration, communication strategies, and training may support wider adoption and engagement.

## Background

Digital Health Technologies (DHTs) encompass a broad range of tools, including mobile health applications (mHealth), patient portals, and electronic health records,^1^^,^^2^ which leverage information and communication technologies to enhance service delivery and accessibility.^3^ These innovations aim to address limitations in traditional healthcare systems, such as geographic barriers and service delays, to improve patient engagement, care coordination, and ultimately patient outcomes. For example, one study of an mHealth diabetes self-management system reported that patients perceived benefits in tracking disease-related measurements, receiving medication reminders, and interacting with providers in a more efficient and informed way.^4^ Similarly, artificial intelligence (AI)-assisted platforms have also shown improvements in patient satisfaction by optimizing the outpatient processes.^5^

Evidence from clinical studies and systematic reviews further supports the potential of DHTs to improve patient outcomes. A systematic review of 13 randomized controlled trials indicated that e-health interventions were effective in improving hemoglobin A1c (HbA1c) control among adults with diabetes mellitus in the short term, although this effect was not maintained at follow-up.^6^ Another systematic review found that interactive eHealth interventions can improve medication adherence among adults using long-term medication, particularly when interventions teach medication management skills, coordinate adherence care, or facilitate patient-provider communication and decision-making.^7^ However, evidence for sustained or downstream clinical benefits remains limited, as improvements may not be maintained over time and long-term engagement with mHealth applications can decline after initial adoption.^6^^,^^8^ Moreover, successful implementation of DHTs requires consideration of multiple stakeholders, including patients, caregivers, healthcare providers, and policymakers, because adoption may be affected when stakeholder needs and expectations are not adequately considered or aligned.^9–11^ Yet, much of the existing literature has examined DHT adoption from specific stakeholder perspectives, such as healthcare professionals or older adult patients, rather than comparing perspectives across multiple stakeholder groups.^9^^,^^12^^,^^13^ This gap in understanding may contribute to lower adoption or implementation failure.^10^^,^^11^

In Hong Kong, a dual-track healthcare system consisting of both public and private sectors^14^ has been adopted for decades to deliver efficient and high-quality health services. This is reflected in strong health indicators, such as infant mortality rate of 1.6 per 1000 registered live births and a maternal mortality ratio of 3.0 per 100 000 registered live births in 2023.^15^ Both indicators remain well below recent global estimates reported by the World Bank and World Health Organization.^16^^,^^17^ However, the public sector bears a disproportionate share of the service burden, accounting for 90% of inpatient care and nearly all emergency services.^14^^,^^18^ This imbalance places increasing strain on the public system, particularly in the context of Hong Kong’s rapidly aging population and increasing chronic disease burden.^19–22^ To address these challenges, the Hong Kong Hospital Authority (HA) launched HA Go in 2019,^23^ a one-stop mobile health system navigation tool designed to improve patients’ experience when using public hospital services and support their health management. The platform provides a wide range of functions including checking appointments, paying bills and drug charges, booking appointments for general and specialist services, viewing medication details and following rehabilitation exercise instructions.^24^ By empowering patients to manage their health more proactively, HA Go may support more convenient access to healthcare information and facilitate personal health management.^24^

Despite its potential, no studies to date have assessed the perceptions of HA Go’s key stakeholders regarding the platform’s implementation and perceived usefulness. Considering the broader gaps in DHT research—particularly the lack of multi-stakeholder perspectives—this study aimed to explore the enablers and barriers to HA Go adoption among users, non-users, caregivers, physicians, and policymakers. It also sought to identify variations in perceptions across stakeholder groups to inform more inclusive and effective digital health strategies.

## Methods

### Study design

This qualitative study was conducted via online individual interviews with key stakeholders. Ethical approval was obtained from the Survey and Behavioural Research Ethics Committee, The Chinese University of Hong Kong (SBRE-23-0841). The study objectives and the interview logistics were explained to the study participants before written informed consent was obtained. Written informed consent was obtained from participants, with assurances of confidentiality. An incentive in the form of a shopping coupon valued at HK$100 was provided to each participant after the interviews to recognize their time and contribution to the completion of this study. The research was conducted in accordance with ethical guidelines, following the Declaration of Helsinki. The Consolidated Criteria for Reporting Qualitative Research (COREQ) checklist guided the reporting of this study (Appendix).^25^

### Study subjects

Six stakeholder groups were invited to participate in this study to explore their attitudes, perceptions, enablers, and barriers related to the installation and adoption of HA Go. These groups included: (1) patients who had used HA Go in either public or private healthcare settings (users); (2) patients who had not used HA Go (non-users); (3) caregivers of patients in Hong Kong; (4) physicians working in public hospitals or non-governmental organizations; (5) physicians practicing in the private sector; and (6) policymakers and health informatics managers involved in the design, maintenance, or potential modification of HA Go. Participant recruitment was guided by the principle of data saturation, defined as the point at which successive interviews yielded no substantively new themes and additional data resulted in minimal or no changes to the coding framework or codebook.^26^ We aimed to recruit approximately five participants per group to ensure representation, while conducting additional interviews as needed until within-group saturation was achieved. This approach is consistent with methodological guidance on data saturation and prior Consolidated Framework for Implementation Research (CFIR)-guided multi-stakeholder qualitative research.^26^^,^^27^

### Subject recruitment

Purposive sampling was employed to ensure a diverse representation of stakeholders from different groups.^28^ Patients and caregivers were approached directly in clinical settings, specifically at the Lek Yuen General Out-patient Clinic. Physicians were recruited through a list provided by the Hong Kong Medical Association and referrals from the Principal Investigator’s social network. Policymakers and health informatics managers were identified through professional networks. Among the stakeholders approached, one eligible participant did not complete participation due to organizational operational factors.

### Data collection

Each interview lasted approximately 30–60 minutes and was conducted via Zoom or phone calls. A semi-structured interview guide was developed to examine attitudes, perceptions, enablers, and barriers regarding the installation and adoption of HA Go. Prior to formal data collection, the interview guide and the moderator’s manual were pilot-tested with interviewees from various groups. Insights gained from the pilot interviews were used to refine the questions for improved clarity and contextual appropriateness. During the interviews, the finalized version of the guide was implemented, with additional follow-up questions incorporated as new themes emerged. Two trained research assistants (CYL and XZ) conducted all interviews. One served as the facilitator and the other as the observer, and both had prior experience in qualitative data collection. All interviews were audio-recorded, with facilitators making field notes throughout each interview.

### Data processing and analysis

Audio recordings were transcribed verbatim using standardized procedures, and transcription accuracy was verified by an independent researcher. Data were managed and analyzed using NVivo 12 (QSR International) and Microsoft Excel. Analysis was conducted using the original Chinese transcripts, and quotations were translated into English for reporting; interview notes were consulted to resolve ambiguities. Translation accuracy was subsequently checked and confirmed by a senior researcher. Each transcript was coded to identify key content areas, and narrative themes were developed through close examination of the data. Coding was conducted independently by two researchers, with discrepancies resolved through discussion and consensus.

Data analysis was conducted in two phases that combined thematic analysis with CFIR-guided interpretation. In the first phase, inductive analysis was conducted to generate initial codes and identify emerging patterns and preliminary themes from participants’ accounts. In the second phase, these preliminary themes were systematically revisited and refined using the CFIR as a guiding framework, which includes five domains: Innovation, Outer Setting, Inner Setting, Individuals, and Implementation Process.^29^ Both barriers and facilitators were further categorized within these domains to provide a structured and theory-informed understanding of stakeholder perspectives on HA Go implementation. Findings were reported using CFIR domains and constructs as an organizing structure, with themes and illustrative quotations presented within each domain.

### Quality assurance

Interviewers received standardized training to ensure competence in qualitative interviewing, consistent application of the CFIR framework, and uniform implementation across interviews. Training covered CFIR concepts, interviewing techniques, ethical procedures, practice interviews, and cultural sensitivity. To safeguard data quality, a structured interview protocol was developed to guide pre-interview preparation and interview conduct. A senior researcher (CCZ) periodically observed interviews to monitor adherence to the protocol and provide ongoing feedback.

## Results

### Characteristics of participants

A total of 33 participants were included in this study, including 15 from the group of users, non-users, and caregivers, 13 physicians, and 5 policymakers involved in the development or management of HA Go. All participants in the user, non-user, and caregiver groups were aged over 60 years. The majority were female (80.0%, *n* = 12), had a monthly household income below HK$15,000 (60.0%, *n* = 9), and reported having at least one chronic disease (86.7%, *n* = 13) (Table 1). Socio-demographic characteristics of the physician group are presented in Table 2. Among the 13 physicians, 61.5% (*n* = 8) practiced in the public sector, 84.6% (*n* = 11) were specialists in family medicine, and 76.9% (*n* = 10) were fellows of the Hong Kong Academy of Medicine. Seven physicians had clinical experience of over 15 years, and the largest proportion of physicians (46.2%, *n* = 6) reported daily patient volume over 50 patients. Details of the policymaker group are summarized in Table 3. Most were female (60.0%, *n* = 3), aged between 51 and 55 years (40.0%, *n* = 2), and held the position of Chief Manager within HA (40.0%, *n* = 2). The majority had been in their current role for 3 to 6 years (60.0%, *n* = 3), and all reported being very familiar with the HA Go application.

**Table 1.** Demographic characteristics of users, non-users, and caregivers (n=15).

Demographic characteristics	Total number (Percentage) n (%)
**1. Sex**	
Male	3 (20.0%)
Female	12 (80.0%)
**2. Age**	
>60	15 (100.0%)
**3. Education level**	
Primary school	5 (33.3%)
Secondary school	8 (53.3%)
Tertiary education or above	2 (13.3%)
**4. Monthly household income (HKD)**	
<HK$15000	9 (60.0%)
HK$15000-HK$30000	6 (40.0%)
**5. Chronic disease(s)**	
None	2 (13.3%)
1	6 (40.0%)
>1	7 (46.7%)

**Table 2.** Characteristics of physicians (n=13).

Demographic characteristics	Total number (Percentage) n (%)
1. Sex	
Male	6 (46.2%)
Female	7 (53.8%)
2. Age (years)	
31-35	2 (15.4%)
36-40	5 (38.4%)
41-45	2 (15.4%)
46-50	2 (15.4%)
51-55	2 (15.4%)
3. Practice experience (years)	
6-10	1 (7.7%)
11-15	5 (38.4%)
16-20	2 (15.4%)
21-25	3 (23.1%)
26-30	2 (15.4%)
4. Training status	
Nil	1 (7.7%)
Higher trainee	1 (7.7%)
Completed higher training	1 (7.7%)
Academy fellow	10 (76.9%)
5. Practice setting	
Public	8 (61.5%)
5.1 Practice type	
FMC	8 (61.5%)
Private	5 (38.5%)
5.2 Practice type	
HMOs	2 (15.4%)
Partnership	3 (23.1%)
6. Specialty	
Nil	1 (7.7%)
Family medicine	11 (84.6%)
Orthopaedics	1 (7.7%)
7. Practice size (number of consultations daily)	
10-29	5 (38.5%)
30-49	2 (15.4%)
50 and above	6 (46.2%)

Abbreviations: FMC: Family Medicine Clinics (formerly known as General Out-patient Clinics); HMOs: Health Maintenance Organizations.

**Table 3.** Characteristics of policymakers (n=5).

Demographic characteristics	Total number (Percentage) n (%)
1. Sex	
Male	2 (40.0%)
Female	3 (60.0%)
2. Age (years)	
36-40	1 (20.0%)
41-45	1 (20.0%)
46-50	0 (0.0%)
51-55	2 (40.0%)
56-60	1 (20.0%)
3. Current title in HA	
User group chairman	1 (20.0%)
Chief manager	2 (40.0%)
Senior health informatician	1 (20.0%)
Administrator	1 (20.0%)
4. Duration of employment in current position (years)	
<3	2 (40.0%)
3-6	3 (60.0%)
5. Familiarity with HA Go application	
Very familiar	5 (100.0%)

Abbreviation: HA: Hospital Authority.

### Barriers and facilitators to the adoption of HA Go

Key themes emerged from the interviews and were categorized according to the CFIR domains of Innovation, Outer Setting, Inner Setting, Individuals and Implementation Process, reflecting the perspectives of users, non-users, caregivers, physicians, and policymakers. Tables 4 and 5 present the barriers and facilitators identified among users, non-users, and caregivers, and among physicians and policymakers, respectively, together with representative quotations for each theme. The following sections summarize the themes by CFIR domain and construct, with particular emphasis on the major facilitators and barriers and the potential relationships between them.

**Table 4.** Barriers and facilitators to the adoption of HA Go among users, non-users, and caregivers guided by CFIR framework.

CFIR domains & constructs	Quotations of facilitators	Quotations of barriers
Innovation—source		
	**Government source as a facilitator of trust (*n* = 2)** *“HA Go belongs to The Government so I do have confidence.” [CG004]*	
Innovation—relative advantage		
	***Facilitating patients’ appointment management (n = 11)*** *“If I tell the system that I need to go to my follow-up tomorrow, then it will tell me when I have it and what time any other appointments I have.” [UP001]* *“I have tried making an appointment to see the doctor once. It was pretty good and convenient. Like you can see the different clinics and such so you don’t need to call and go through that hassle.” [UP005]*	**Not useful in geriatric care (*n* = 2)** *“Well, and especially since you’re talking about the elderly care aspect, I’ve had enough. I don’t feel like there’s anything in particular that’s helpful… I would even say that it’s not enough” [CG001]*
	**Smoother healthcare service journey by simplified process (*n* = 9)** *“I directly scan the referral letter and afterwards I’ll send it to them and patiently wait for them. If the hospital says they haven’t received it, they will send back a letter and make an appointment on a random date for me. I think this is very good.” [CG004]* *“No need to queue up…that means it won’t take too much time” [CG002]* *“Paying within 2 hours before is so convenient. You don’t have to queue.” [UP003]*	
	**Improvement in physician-patient communication (*n* = 2)** *“There are sometimes where we ask them if they would talk about it with the doctor. Tell them to bring the report. When they go to see the doctor, they have completely forgotten about this or don’t know how to ask. Well now that there is this report, it has made it better… It doesn’t take too long for the information to be added.” [CG004]*	
	**Improved access to medical records** (*n* = 5) *“I mean now with a click of a button; you can record what medications the doctor has prescribed.” [UP005]* *“And viewing those things, I mean after drawing blood, etc. those reports.” [UP003]*	
Innovation—complexity		
	-	**Complexity of use (*n* = 5)** “*Like at the moment, the system has guides and instructions on there and after reading it, I don’t really understand. I don’t even know how to click into it. It would be better if the app were more convenient and user-friendly and not so complicated.” [NU001]* *“The worst part is that it really is comparatively complicated. As a result, the elderly doesn’t really know how to use it.” [UP003]*
		**Unnecessary registration steps (*n* = 1)** *“Some people feel that going through the authentication process is troublesome… Initially, you need to place your ID card properly and have it scanned by the machine to determine whether you’re a real person or not. Eventually, it seems like you need to go to a hospital or clinic counter, where you have to show up in person, and they press a button or something like that. This adds an extra step.” [UP002]*
		**Complex appointment process for general outpatient services (*n* = 1)** *“On that day, if you have something in particular that you want to get checked out then you wouldn’t be seeing a specialist…Well, I think this is a hindrance.” [UP002]*
Innovation—design		
	**Functional improvements (*n* = 6)** *“I think that it would be best… For example, if I have a doctor’s appointment tomorrow, even though the system will remind us, sometimes, just listening to it is not good enough. The best would be if had an alarm or so instead. I think that this would be better” [UP001]* *“If there are any activities or things to help us elderly folks with cognitive impairments, or anything else, then hopefully it can help us or provide us with cognitive or physical activities to improve our memory.” [NU004]* *“It’d be better to add more of these informational functions—give us more access.” [CG002]*	**User interface issues (*n* = 4)** *“I think their notifications are rather annoying. Like right after registering, you will get a notification. After it notifies you, you will then need to click it and log in again. After registering, there are several lines of notifications with red dots that you will need to click it and it will pop out again.” [UP005]*
	**User interface improvement (n = 5)** *“Earlier I was emphasizing—I really want your booking system to be clearer and more visible. The future appointment schedule should be instantly recognizable… I need this to show very clear timings.” [CG004]*	
	**Clear function design (*n* = 4)** *“Um, I think it is pretty clear. Like if there’s anything, you can click into the system and very clearly explain it to you and I think this is pretty good.” [UP001]* *“I’m satisfied that it’s so clear. The pictures have a bit of directions to show you so that you understand what it’s talking about and which app is for what.” [CG005]*	
	**Platform separation needed (*n* = 1)** *“As in, if those things are separated into another platform, I’m talking about something like having the Hospital Authority’s health education separated into another platform, it would be better because it wouldn’t be stuffed into HA Go App since it is primarily meant to view your own information.” [UP005]*	
Individuals—need		
		**Perceived lack of need for HA Go (*n* = 4)** *“It seems like at the moment, I don’t need it. Because for now, I don’t need to go see a doctor.” [NU003]* *“Because my dad and mom, etc. would often use it and directly press the medical alert button or directly go to the follow-ups… All of these things, you need to depend on yourself to go. No need to book in advance.” [NU005]*
Individuals—capability		
		**Knowledge and familiarity of digital devices (*n* = 9)** *“I don’t know how to use the phone or press the button either… And after playing for a while, it redirected to another section or I’ve pressed the wrong button.” [UP002]* *“I’m not one of those people who are very familiar with it. I won’t do so much because I can’t handle it. I can’t remember so many things.” [UP005]* *“I don’t know how to use it, so I don’t use it (HA Go) (NU004)”*
		**Functional and health-related barriers (*n* = 3)** *“Some people really have limited mobility. I think it is hard for them because I know these people. As in, their fingers are not very agile.” [UP002]*
Individuals—motivation		
		**Personal preference (*n* = 4)** “*Like unless sometimes when you’re suddenly sick, you wouldn’t think about using HA Go to book an outpatient appointment or those things. Instead, I would go to see a private doctor.” [NU001]* *“Since I have joined a senior center, their information is very updated so if they know something, as in those treatment vouchers for public use or something like that, I would find out from there. Like, it’s not necessary for the health bureau to pass down the news to me.” [NU002]*
	**Trust in privacy protection (*n* = 1)** *“eHealth, HA Go, and other online applications are safe to use of course. I believe it is safe so I’m very at ease.” [UP003]*	**Concerns about data security (*n* = 5)** *“The most important thing is… also need to verify the IDs. As in afterwards, you still need to take a picture of your ID and place it correctly when you’re taking the picture… I’ve heard a lot of people are very afraid of it.” [UP002]* *“What I’m actually concerned about is that there will be a data leak.” [NU002]*
		**Low motivation to learn and use (*n* = 2)** *“Like if that person doesn’t want to learn new things and just sit around at home or something. Sitting there, there’s the phone with the dial and you use your fingers to dial, that type of person. Then it doesn’t work. Then to also make an appointment to ask someone, they would also not be willing.” [UP002]* *“I’m rather opposed. Honestly, in regards to these types of apps, learning now, you would have to waste a lot of time online to search for the information about how to use them…” [UP005]*
Individuals—opportunity		
	**External influences on patient decisions (*n* = 7)** *“ I also have some friends who use it. They say seeing the doctor is very convenient. Don’t need to call and keep pressing the keypad just to end up not finding the right person to talk to.” [UP005]* *“These family members and some healthcare personnel require me to use it. I used it. It (HA Go) provided convenience for us.” [NU004]*	**Device and internet issues (*n* = 3)** *“Unless my phone runs out of battery. If your phone runs out of battery then there’s nothing you can do.” [UP004]* *“I can’t use the Wi-Fi to update. I use my own data to update but if it doesn’t work… Going to HA Go’s Wi-Fi, I also need to use my own data… First of all, it wastes my data and second of all, it’s not like the regular Wi-Fi outside where you can go online and see things for what they are. I don’t like it so much, really go to destroy it.” [UP005]* *“I don’t really like it. It’s only my own problems. Since I have already lost a few of my phones, your things have all been set on there. Moreover, I am the caretaker. My mom and my little brother, their information is on there, so I wouldn’t. Those things are completely lost. Reporting to the police isn’t useful anyways.” [NU002]*
	**Availability of time to explore HA Go (*n* = 1)** *“More normally, it will be very simple or if there’s something that comes up, I would go take a look.” [UP001]*	**Competing time demands limiting HA Go usage (*n* = 2)** *“Because now I don’t have the time to do these things. Since I have two elderly people I have to watch over.” [NU005]*
Inner setting—incentive systems		
	**Rewards encouraging use (*n* = 2)** *“They previously gave me a small present but it wasn’t appealing. Afterwards, they gave me another one that is for putting your phone on. I think that it’s somewhat useful and it appealed to me.” [UP005]* *“I think at the very beginning, there was a gift. It was some sort of calendar or something to be gifted, I think. Like it would lead you to discover these things.” [UP002]*	
Inner setting—access to knowledge & information		
	**Availability of knowledge resources for patients and healthcare professionals (*n* = 13)** *“Because in the hospital, sometimes they will help people with it. And I originally had joined the Love your Health Association- those Health Associations- and the social worker would help.” [UP003]* *“There is a teacher after all. If I stumble across any difficulties, I can give him/her a call or go to ask him/her. Well, he/she is pretty good and will help us.” [UP004]*	**Insufficient knowledge resources and support (*n* = 1)** *“Now, it is difficult. The volunteer group is no longer around but honestly speaking, at that time, the volunteer group only helped you install it. If you asked more questions, they wouldn’t know how to respond.” [UP005]*
	**Training and education required (*n* = 4)** *“The best is for it to be more universalized or they can hold a class. I mean start a class to teach those who really don’t know how to use it or those who are afraid to use it.” [UP002]*	
Process—engaging		
	**Effective patient promotion and outreach (*n* = 1)** *“There should be someone to promote it. Like, at least in places like clinics, there should be some- I mean publishing notices or individuals promoting it, something like that.” [UP002]*	**Lack of patient outreach and promotion (*n* = 1)** *“I don’t really feel like HA Go developer has done any promotion in that area, or maybe I just haven’t come across it. Hmm. Like on TV, or on the radio, in newspapers, or anything like that—it seems like I don’t see much of it.” [CG001]*
Process—adapting		
	**Timely update and improvement needed (*n* = 1)** *“If in the future it’s possible for you to keep developing it, then there would be lots of things that are very good. And it would change and become different. As in it would be more convenient for me. Well in this aspect, I would also be interested in it.” [CG001]*	

Abbreviations: CFIR: Consolidated Framework for Implementation Research; UP: user participant; NU: non-user participant; CG: caregiver.

**Table 5.** Barriers and facilitators to the adoption of HA Go among physicians and policymakers guided by CFIR framework.

CFIR domains & constructs	Facilitators	Barriers
Innovation—relative advantage		
	**Facilitating patients’ appointment management (*n* = 16)** *“It would remind them when they have an appointment because sometimes if it’s been too long ago then they would forget.” [PV001]* *“At least there would be someone to remind you. Then you wouldn’t need to look through so many papers.” [PB002]* *“They can use HA Go to make bookings or reschedule appointments. Well, this is definitely doable.” [SH003]*	**Access to health data causes patients’ confusion (*n* = 3)** *“There’s more information. Maybe it will lead to them knowing before I explain to them. It could lead to increasing their anxiety.” [PB003]* *“Sometimes there are some patients who, after looking at the information, may not completely understand it. There was a patient that had a blood test… Actually, his/her report was completely normal and had no issues… but because he/she was using his/her phone to view it, they saw this particular section and thought that it was talking about his/her current state. Then after, he/she completely misunderstood the entire report’s meaning.” [PB004]*
	**Smoother healthcare service journey by simplified process (*n* = 17)** *“Their process, like if they do other things, like pick up medication, make a payment, or check-in, everything is smoother.” [PB002]* *“Less queuing, less waiting… It’s convenient…. You can pay in advance by electronic payment… It’s so fast and smooth.” [PV002]* *“Because HA Go itself has made a lot of progress in clinics, the whole system has improved the patient journey, especially in outpatient clinics… it has also been helpful for inpatient services. It has increased the efficiency for patients.” [SH003]*	–
	**Usefulness for patient medication management (*n* = 13)** *“I think a lot of the patients in Hong Kong don’t know what medications they are taking so if it has a record for them, then they will know and it would be better for them.” [PV001]* *“I think it serves as a reminder… for example, if a patient is taking medication but sometimes forgets the dosage or the correct way to take it… they can actually check their prescription in the system. So, through the system, they can look up the correct dosage and method for taking their medication.” [PB004]* *“For example, with medications, we have a barcode that can be scanned, but now you don’t even need to scan it. You just need to click on the medication you are taking, and all the side effects will be displayed for you. “[SH001]*	
	**Improvement in health management contributed by patient empowerment (*n* = 16)** *“There is some patient information, exercise materials, etc. provided through HA website, and it has been checked by the Hospital Authority so the patients can have confidence and use it. Well, this is a good thing.” [PB002]* *“Because it includes some topic-based information that can serve as a reference for different types of patients. I’ve noticed that it’s quite detailed, especially when it comes to things like postpartum care for children, nutrition, and various other aspects. There’s actually a lot of information available.” [PV003]* *“HA Go provides a platform for patients to access more information to take care of themselves, and their families can also learn to care for themselves.” [SH001]*	
	**Enhancement of caregiving quality by caregiver-specific features (*n* = 9)** “*it can help in patients caring, it has a function about carer…they can access to patient’s (information)…beneficial for patient support (PB008).” [PB008]* *“Now there’s the ‘My Friends and Family’ feature. You can take care of your children, care for the elderly, or even share it with your family members to help you take care of them, or with a domestic helper.” [SH001]*	
	**Improvement in physician-patient communication (*n* = 10)** *“The patient has already made a reservation. Perhaps when they come, they have already stated that they haven’t been controlling what they eat… and they’ve already seen the blood test. For example, that BP record function… Well, it does help the communication between us.” [PB003]* *Patients don’t need to waste time waiting outside the consulting room…… Maybe not so annoyed …. It helps us all to communicate. [PB002]* *I hope that by utilizing this kind of messaging system, healthcare professionals can continuously assist patients by providing them with more information and treatment options. I believe this method can help bridge the gap between everyone, although face-to-face interactions are still invaluable. [SH002]*	
	**Improved resource allocation, continuity and efficiency of medical system (*n* = 15)** *“It can help by enhancing this continuity of care. They can accept the continuous series of treatments. Even if they no longer decide to seek medical attention or treatment at public facilities, the information will be on here and they can still access it.” [PB004]* *“Because actually, if HA Go is linked to both general outpatient and specialty services, the storage of the records and files will be here so it is even more consistent.” [PV002]* *“But now, using this method, the whole society becomes more altruistic. For example, if I (patients) recover, I (patients) can free up my appointment slot for someone else. Or if I (patients) go to a private doctor, I (patients) can release a few slots for others, allowing public resources to be utilized more effectively.” [SH001]*	
	**Improving patients’ adherence and compliance (*n* = 5)** *“It will help (compliance). Because after seeing more information, they will have more faith, I think.” [PV001]* *“If they can have a chart that they can enter and then tick checkboxes on the chart, then it should improve their compliance.” [PB005]*	
	**Environmentally friendly (*n* = 4)** *“In the electronic platform, you can resolve many problems. As a result, there’s less paper used, cash transactions. Like it has become convenient for it to be an environmentally-friendly regulations.” [PB002]* *“And it doesn’t require paper so I think, in terms of environmental conservation, it is a good thing.” [PV002]*	
Innovation—complexity		
		**Complexity of use (*n* = 7)** *“There are some features that can be improved. For example, the remote consultations. If I use HA Go for a video consultation, it is rather a bit difficult to use.” [PB008]* *“After patients finish their outpatient visit, they can use HA Go app to submit prescriptions to the pharmacy. However, patients sometimes submit them too quickly before I finish or make changes. Once submitted, I can’t modify the prescription, which leads to a lot of back and forth, as I have to cancel it at the pharmacy to make changes. This complicates the medication adjustment process.” [PB007]* *“I’m not really clear on how to add caregivers because they need to be authorized. This makes it a bit complicated.” [PV003]*
		**Online queuing creating unpredictability in scheduling (*n* = 2)** *“You can check-in online in advance… But for doctors, we might not find it very convenient because the system isn’t perfect. It allows people not to queue (in person), but there are some patients who check in online, and their names are already on my patient list. However, I often can’t find them.” [PB003]*
Innovation—design		
	**Functional improvements (*n* = 16)** *“And also, for example, post-discharge information—I think this is important. Sometimes we wonder: After leaving the hospital, who should I contact if I have a problem? If I’m discharged from a certain ward, where do I call? After a clinic visit, how do I follow up? These things should be in an app—no need to call or visit in person. But right now, whether for discharged, inpatient, or outpatient cases, it feels like there’s a complete information gap after leaving the hospital.” [SH006]* *“The Hospital Authority should make better use of AI… If you’re still stuck with traditional digital interfaces, you can’t keep up with the times. In fact, many patients are already using AI tools like DeepSeek to look up medical information themselves.” [PV003]* *“In fact, the report just shows all the blood pressures, but there are no analytical features…that can be further optimized…if it can be set a little bit of analytical function (PB008)”* *“The area for improvement is in the interaction. Right now, it’s a bit one-sided; we push information to you. However, in the future, patients could also provide feedback to us, or share their experiences with our frontline staff.” [SH001]*	**User interface issues (*n* = 5)** *“The features inside aren’t very user-friendly. For example, they only allow us to view data from the past 28 days. Sometimes, we want to look further back to make comparisons, but we have to click through and adjust the duration ourselves.” [PB006]* *“The push notifications are quite repetitive and overwhelming. Every time, I have to click on the notification message, but once I see it, I already know what it says. However, it remains on the screen unless I click on each one individually.” [PB001]* *“The interface should be as simple as possible. There are important pieces of information, but everything is crammed into the app. When patients open it, they have to choose from a jumble of options, which can be overwhelming.” [PB002]*
	**User interface improvement (*n* = 9)** *“The fonts and icons should be clear and easy to understand…for older adult…visual impairment making it less convenient for them.” [PB002]* *“For students or young people, managing emotions is important, and this aspect should be prominently featured on the web page. Whether it’s for young people or their caregivers, there should be information or support networks easily accessible. Mental health is a major concern right now, and it’s something society really needs to focus on.” [PV003]* *“One area for improvement is accessibility—currently, many vulnerable groups, not just those in poverty but also the elderly, ethnic minorities, and people with disabilities or visual impairments, have shared feedback. We should explore how HA Go can better accommodate their needs.” [SH003]*	
	**Cross-platform integration needed (*n* = 12)** *“I think it would be worth considering whether to merge HA Go and the eHealth app to combine overlapping features, reducing the number of different apps. This would make it more convenient.” [PV001]* *“Another point is that citizens nowadays place great importance on public-private information sharing. Ensuring interoperability would be beneficial, and in the long term, there could even be cross-border or Mainland interoperability.” [SH003]*	
Individuals—need		
	**Perceived usefulness in meeting healthcare management needs (*n* = 2)** *“But if you’re someone with a chronic illness who has frequent follow-up appointments, or if you need to take care of your family members, then there’s a lot to keep track of.” [SH001]*	**Perceived lack of need for HA Go (*n* = 5)** “*There are some people who have never used public services, so they don’t need the app. [PV002]* *“It depends on whether they feel the need for it. Some people might already think that the whole process is convenient or easy to manage, so they may not feel the need to specifically download the app.” [PB007]*
Individuals—capability		
		**Knowledge and familiarity of digital devices (*n* = 14)** *If they are not very familiar with these electronic systems, older individuals may lack the confidence to use the apps. It mainly has to do with their age. [PV001]* *Age is definitely related, as learning new things tends to be slower with age. [PB002]* *I believe that age is a factor, and older adults may find it more difficult to adapt. [SH003]*
		**Functional and health-related barriers (*n* = 6)** *“If patients have difficulties reading text, they may not know many characters, which can make using the app challenging. Even if they can read, they might have visual impairments that make it hard to see, leading to these difficulties.” [PV004]* *“Unlike generally healthy citizens who are agile and can easily use other electronic platforms, some patients may have conditions that make their hands and feet less nimble. This affects their ability to use the app independently.” [PB002]* *“Those elderly who are too old may not be able to handle it, or those with cognitive issues.” [SH001]*
		**Healthcare providers’ familiarity with HA Go (*n* = 2)** *“Another factor is that healthcare professionals need to be more familiar with the app and aware of its use, which can enhance its practicality.” [PB008]* *“But I actually feel that, to be honest, even many of our colleagues may not be aware of the new functions sometimes. If they don’t know, then they can’t help promote or explain them to the patients, which reduces their impact. So, I think, in this regard, how can we enable colleagues to gain knowledge about this?” [SH006]*
Individuals—motivation		
	**Motivation to use HA Go (*n* = 1)** *“(Patients) really want to take control of my own information and my own medical condition, right?” [SH001]*	**Resistance to use HA Go (*n* = 2)** *“The limitation lies in whether they accept this new approach. People are used to certain patterns, and shifting to a different one can be a significant barrier. The habits of individuals themselves can restrict this change.” [PB002]*
		**Worries about operation errors (*n* = 2)** *“Older individuals may have less confidence in using platforms for tasks like making payments or booking appointments, as they fear making errors in scanning or booking. Therefore, they may prefer to handle things in person.” [PV001]*
		**Limited motivation due to unfamiliarity with digital devices and interface (*n* = 3)** *“In other words, their willingness to use it means that if some older individuals cannot find what they want after pressing a few buttons, they are likely to stop using it and give up.” [PB004]*
		**Perspectives on privacy protection (*n* = 6)** *“Ultimately, downloading the app involves issues of privacy.” [PB007]* *“Because this mobile app requires verification of personal information like identity card numbers, some patients have concerns about privacy. They worry that if their phone is stolen or lost, their personal data could be exposed. Therefore, privacy is a significant concern for patients and a factor they consider.” [PB004]*
Individuals—opportunity		
	**Influence and assistance from others (*n* = 4)** *“It might be the influence of their family members or peers. Many of these mobile apps are often promoted through word of mouth, meaning someone needs to tell them about the app and its features for them to know.” [PB004]*	**Availability of time to use in private and public healthcare settings** **(*n* = 2)** *“The consultation time is always limited. So, using an application like HA Go to provide pamphlets or to view information about things like blood pressure at home… as far as I’m aware, our colleagues don’t really use it that much.” [PB008]* *“Right now, in my private practice, the environment of the clinic has time constraints, so I actually have less time to spare. I think using this app to help me find the information I wouldn’t have a significant impact.” [PV004]*
Outer setting—local conditions		
	**Inevitability of digitalization (*n* = 2)** *“We are now entering a digital age, and a lot of things should be paperless. This is an inevitable trend in development. Many Government departments and healthcare organizations already have electronic platforms to handle many tasks. Therefore, especially in our healthcare sector, with HA being such a large organization involving so many users, having its own platform is definitely necessary.” [PB002]*	
Inner setting—compatibility		
	**Compatibility with existing medical system (*n* = 7)** *I actually think that it is a platform designed to utilize the healthcare services of our Hospital Authority, and it provides a good support system. [PB002]* *Secondly, regarding government policy data, we have also achieved interoperability with the current eHealth app. [SH003]*	**Compatibility with existing medical system (*n* = 1)** *“If HA Go is said to be responsible for one-way communication with patients, that’s not entirely accurate… It’s actually two-way now. For example, as mentioned earlier, with biometric measurements, patients can input data. However, at the moment, it’s still not fully integrated—unlike some systems where the data can be inserted directly into what we call the CMS.” [SH006]*
		**Limited impact on actual workflow (*n* = 6)** *“To be honest, I don’t think it’s particularly helpful. Because I can access e-health from my own hospital, I don’t see what additional information HA Go can provide me.” [PV002]* *“Our CMS system is still more useful and powerful. Therefore, the information I can access there is much more than what HA Go provides. So, for healthcare professionals, this isn’t necessarily the most convenient option.” [PB008]*
Inner setting—available resources		
		**Lack of resources (*n* = 3)** *“It’s not enough; we really need additional clinical staff. We need extra manpower at our clinics to promote the app and teach patients how to use it. If we want to promote it effectively or have more people using it, we need this manpower.” [PB006]*
Inner setting—access to knowledge & information		
	**Accessible knowledge resources and educational support (*n* = 4)** *“For patients, we have a webpage dedicated to HA Go, where we post short videos and PDFs that provide step-by-step instructions on how to use the app. It also highlights the new key features of HA Go, and we promote those there. We provide links back to the webpage for them to view.” [SH004]*	**Limited access to knowledge resources and training (*n* = 9)** *“If my patients at the Government clinics are unaware of the existence of this app, I think that’s not enough. They might need reception staff or booths to teach people how to use it.” [PV002]* *“In terms of healthcare staff, there may actually be a shortage. We may have to rely on emails, which include attachments and menus, but in reality, not all healthcare personnel can understand how to use it just from an email.” [PB008]*
Process-teaming		
	**Cross-department collaboration required for advocacy (*n* = 3)** *“Of course, this needs to involve discussions with some primary care stakeholders on how to achieve this. I think it’s beneficial.” [SH002]*	
Process—assessing context		
		**Perceived barriers and risks for implementation (*n* = 2)** *“The biggest risks and challenges, I think, relate to carers. It’s important for patients to know how to invite their carers. We need to prevent elderly individuals from being scammed.” [SH001]* *“If a patient has an important report, and we ask them to upload it, do we have sufficient support to monitor all incoming reports 24/7? This would be necessary to ensure we make accurate and reliable decisions, which could be quite challenging.” [SH002]*
Process—engaging		
	**Improvement in publicity strategies (*n* = 11)** *“If the Hospital Authority had more promotion, it would make more patients aware that such an app exists, which would likely increase its usage frequency.” [PB004]* *“I think there should be a bit more advertising or campaigns to inform patients about what HA Go can do.” [PV001]* *“We believe it’s better to leverage more community-based channels for promotion… diverse outreach methods matter.” [SH001]*	**Insufficient promotion of HA Go’s existence and functions for patients and physicians (*n* = 1)** *“Promotion and accessibility support are not enough.” [PV002]*
Process—reflecting & evaluating		
		**Need for feedback loops (*n* = 6)** *“However, we have previously reported that IT doesn’t seem to have implemented many improvements yet, so this is currently frozen.” [PB003]* *“Overall, regarding the experiences patients encounter, it would be ideal to have more platforms for the public and frontline healthcare staff to provide feedback for optimization.” [PB008]*

Abbreviations: CFIR: Consolidated Framework for Implementation Research; PB: public-sector physician; PV: private-sector physician; SH: policymaker or health informatics stakeholder.

## Innovation

### Innovation—source

#### Government source as a facilitator of trust

Some users and caregivers expressed greater confidence in HA Go because it “belongs to The Government” [CG004] suggesting that the official source increased their trust in the platform (Table 4).

### Innovation—relative advantage

#### Facilitating patients’ appointment management

Participants across stakeholder groups highlighted the role of HA Go in facilitating appointment management, particularly in improving accessibility and convenience (Tables 4 and 5). One user pointed out that “*I have tried making an appointment… you don’t need to call and go through that hassle* ” [UP005]. Similarly, physicians and policymakers emphasized the system’s function in supporting appointment adherence and flexibility. For example, one physician remarked, “*It would remind them…because sometimes…they would forget* ” [PV001], while a policymaker noted, “*They can use HA Go to make bookings or reschedule… is definitely doable* ” [SH003]. Overall, participants viewed HA Go as a tool that could reduce the burden of appointment management through reminder functions and flexible booking options.

#### Smoother healthcare service journey by simplified process

Across stakeholder groups, participants described HA Go as facilitating a smoother healthcare experience by simplifying administrative processes (Tables 4 and 5). Users and caregivers particularly highlighted reduced waiting time through advance payment and avoidance of queuing, such as being able to “*pay within two hours before*” and “*don’t have to queue*” [UP003], as well as feeling that the process “*won’t take too much time*” [CG002]. Similar views were also expressed by physicians and policymakers, as a physician in the private setting pointed out that *“less queuing, less waiting…you (patients) can pay in advance…it’s fast and smooth"* [PV002]*,* which emphasized the benefits of HA Go in terms of improving the patient experience as well as increasing the efficiency of service delivery.

#### Improvement in physician-patient communication

Beyond simplifying the healthcare service journey, a shared view across stakeholder groups was that HA Go facilitated communication with doctors by helping patients “*bring the report*” to consultations [CG004] (Table 4). Physicians similarly highlighted that patients could attend consultations after having “*seen the blood test*” and that HA Go could “*help the communication between us*” [PB003]. Policymakers also suggested that the platform could “*bridge the gap*” between patients and healthcare professionals by supporting ongoing information exchange [SH002] (Table 5).

#### Improved access to medical records

In addition to facilitating physician-patient communication, participants in the user, non-user, and caregiver groups further highlighted HA Go as allowing easier access to medical records. Users described being able to review information “*with a click of a button*” including what medications had been prescribed [UP005], as well as test “*reports*” [UP003]. These findings suggested that HA Go was valued for making relevant medical information more readily accessible to users and caregivers **(**Table 4**)**.

#### Usefulness for patient medication management

From the perspectives of physicians and policymakers, HA Go was also perceived as useful in supporting patient medication management (Table 5). They believed that adopting HA Go would assist patients with medication management, such as reviewing prescriptions and identifying the correct dosage and method of administration. For example, a physician noted that the platform allowed patients to review prescription information and served as a reminder when they forgot the “*dosage*” or the “*correct way*” to take their medications [PB004].

#### Improvement in health management through patient empowerment

Moreover, physicians and policymakers expressed that patients’ understanding of their health information, such as illness information and medical reports, would help improve their knowledge of their health and empower them to practice self-care (Table 5). As one policymaker noted, “*HA Go provides a platform for patients to access more information…to care for themselves*” [SH001]. Both physicians and policymakers anticipated that more information related to patients’ illnesses would help strengthen patients’ sense of self-management.

#### Enhancement of caregiving quality by caregiver-specific features

Extending this role in self-management, physicians and policymakers also noted the special role of HA Go for caregivers. As one physician indicated, “*it can help in patients caring, it has a function about carer…they can access to patient’s (information)…beneficial for patient support*” [PB008]. HA Go’s caregiver-specific features, such as managing and accessing the care recipient’s health records, were recognized as potentially improving the quality of caregiving services (Table 5).

#### Improved resource allocation, continuity and efficiency of the healthcare system

Besides highlighting the benefits of HA Go for individual patients, physicians and policymakers also noted improvements in healthcare system efficiency resulting from HA Go adoption (Table 5). They consistently emphasized that the continuity of healthcare services and the efficient use of resources, such as appointment slots, were improved via HA Go’s clinical data sharing and flexible appointment management. For instance, one policymaker noted, *“Now, using this method… I (patients) can free up my appointment slot….if I (patients) go to a private doctor… allowing public resources to be utilized more effectively”* [SH001].

#### Improving patients’ adherence and compliance

Physicians and policymakers further suggested that HA Go could improve patients’ adherence and compliance by making health information and self-monitoring more accessible (Table 5). They noted that greater access to information could strengthen patients’ “*faith*” in treatment [PV001], while functions such as a chart with “*tick checkboxes*” might help patients track their actions more clearly and “*improve their compliance*” [PB005].

#### Environmentally friendly

Physicians and policymakers also viewed HA Go as environmentally friendly because it reduced reliance on paper-based and cash-based processes (Table 5). They noted that, through an “*electronic platform*,” many tasks could be handled more conveniently with “*less paper used*” and fewer “*cash transactions*” [PB002]. Similarly, physicians emphasized that the system “*doesn’t require paper*,” which was regarded as beneficial for “*environmental conservation*” [PV002].

#### Not useful in geriatric care

Despite the facilitators described above, some users, non-users, and caregivers questioned whether HA Go was sufficiently useful for geriatric care **(**Table 4**)**. A caregiver suggested that for the “*elderly care aspect,*” the app was “*not enough*” [CG001]. This indicated that the perceived relative advantage of HA Go may be limited when its functions do not adequately address the more specific needs of older adults.

#### Access to health data causes patients’ confusion

Another barrier identified in the physician and policymaker group was that direct access to health data could sometimes confuse patients (Table 5). Although easier access to information was often seen as beneficial, one physician noted that “*more information*” for patients might “*lead to increasing their anxiety*” [PB003], while another observed that some patients *“may not completely understand”* the information shown in the app and could even *“misunderstand the entire report’s meaning”* [PB004]. These findings suggested that easier access to health data should be accompanied by clearer interpretation to prevent misunderstanding.

### Innovation—complexity

#### Complexity of use

Across all stakeholder groups, complexity of use was identified as a barrier to adoption (Tables 4 and 5). Users and non-users described difficulty understanding the app’s “*guides and instructions*” [NU001] and felt that the system was “*complicated*” [UP003]. Physicians and policymakers also noted that some HA Go functions remained difficult to use. For example, one physician described “*video consultation*” as difficult to use [PB008]. Taken together, these findings suggested that complexity limited the practical value of HA Go at both the user level and the service-delivery level.

#### Unnecessary registration steps

One barrier identified by a user was that the registration and authentication process of HA Go was burdensome and could discourage initial adoption (Table 4). One user described the process as “*troublesome*” and felt that it “*adds an extra step*” before the app could be used [UP002], pointing to the importance of a simpler onboarding process.

#### Complex appointment process for general outpatient services

Beyond the registration stage, another barrier pointed out by a user was that the appointment pathway for general department services could be difficult to navigate through HA Go (Table 4). One user described this as “*a hindrance*” when they had “*something in particular*” to get checked but “*wouldn’t be seeing a specialist*” [UP002], indicating that mismatched appointment pathways may discourage use.

#### Online queuing creating unpredictability in scheduling

At the clinical workflow level, physicians identified online queuing as a barrier when it introduced unpredictability into clinic scheduling (Table 5). One physician noted that patients could check in online in advance, but their names might already appear on the list even when clinicians “*can’t find them*” [PB003], suggesting that online queuing may reduce in-person waiting while creating uncertainty in clinic work flow.

### Innovation—design

#### Functional improvements

All stakeholder groups recognized the perceived need for functional improvements to further enhance the usefulness and adoption of HA Go (Tables 4 and 5). Users, non-users, and caregivers suggested stronger reminder functions such as an “*alarm*” [UP001], more health information with “*more access*” [CG002], and features tailored to older adults, including support for “*cognitive or physical activities*” [NU004]. Physicians and policymakers similarly highlighted the need for more advanced features, including detailed medical history and reporting functions, interaction tools with healthcare professionals or frontline staff, enhanced reminder alarms, and group-specific functionalities tailored to different user needs. One physician pointed out that the report showed blood pressure records but lacked “*analytical features*” and could be “*further optimized*” [PB008]. These observations suggested that incorporating more interpretive and personalized features could strengthen the system’s clinical relevance and improve user engagement with HA Go.

#### User interface improvement

User interface design emerged as an important factor influencing the adoption of HA Go across stakeholder groups (Tables 4 and 5). One caregiver highlighted the need for booking information to be “*clearer and more visible*” *[CG004]*, while physicians and policymakers emphasized clearer “*fonts and icons*” [PB002] and better “*accessibility*” for “*many vulnerable groups*” [SH003]. These findings indicated that improvements in font clarity, visual design, and the reduction of redundant features could enhance usability, particularly for older users.

#### Clear function design

Beyond broader user interface improvements, some users and caregivers also highlighted clear function design as a facilitator of HA Go adoption (Table 4). One caregiver noted that pictures provided “*directions*” to show “*which app is for what*” [CG005]. This suggested that clear labeling and visual guidance might make HA Go easier to navigate and more acceptable to users.

#### Platform separation needed

One user suggested that some content, such as health education information, would be better “*separated into another platform*” rather than being “*stuffed into HA Go App*” [UP005], which may improve the focus and usability of HA Go (Table 4).

#### Cross-platform integration needed

At a broader system level, physicians and policymakers emphasized the need to integrate public and private hospital information-sharing platforms, merge HA Go with other electronic health applications, and reduce overlapping features (Table 5). One physician noted that HA Go and the eHealth app could be merged to “*combine overlapping features*” and make the system “*more convenient*” [PV001]. These views suggested that reducing fragmentation and consolidating platforms might improve usability and convenience for users.

#### User interface issues

Despite these suggested design improvements, participants across stakeholder groups also identified user interface issues as barriers to the adoption of HA Go (Tables 4 and 5). A user described the notifications as “*annoying*” and difficult to manage [UP005]. Some physicians likewise reported that some features were not “*user-friendly,*” [PB006], that notifications could be “*repetitive and overwhelming,*” [PB001] and that information was overly “*crammed into the app*” [PB002]. Overall, these findings suggested that interface-related problems might weaken the accessibility and practical value of HA Go.

## Individuals

### Individuals—need

#### Perceived lack of need for HA Go

Some non-users perceived little need to use HA Go when they were not currently seeking medical care or did not rely on functions that required advance booking (Table 4). One non-user stated that “*I don’t need it*” because there was no current need to “*go see a doctor*” [NU003], which suggested that adoption was less likely when HA Go was not seen as relevant to immediate healthcare needs.

#### Mixed opinions on whether HA Go met the needs

Physicians and policymakers also expressed mixed views on whether HA Go met users’ needs (Table 5). They suggested that the app was more useful when patients had chronic illnesses, frequent follow-up appointments, or caregiving responsibilities, as there was “*a lot to keep track of*” *[SH001]*. However, some people “*don’t need the app*” if they have “*never used public services*” [PV002] or already find existing care processes “*convenient*” and “*easy to manage*” [PB007]. This suggested that the perceived need for HA Go varied according to the complexity of care management.

### Individuals—capability

#### Knowledge and familiarity of digital devices

Across stakeholder groups, limited familiarity with digital devices was identified as a common barrier to the adoption of HA Go (Tables 4 and 5). Users, non-users, and caregivers described difficulties navigating the app, such as pressing the “*wrong button*” [UP002] or simply “*don’t know how to use it*” [NU004]. Physicians and policymakers raised similar concerns, especially for older adults, who were perceived as more likely to “*lack the confidence to use the apps*” [PV001]. These findings suggested that limited digital literacy and confidence might hinder the adoption of HA Go, particularly among older adults.

#### Functional and health-related barriers

Beyond digital familiarity, participants across all stakeholder groups also identified literacy, physical ability, and health conditions as barriers to the adoption of HA Go (Tables 4 and 5). A user mentioned that some people found the app difficult to use because “*their fingers are not very agile*” [UP002]. Physicians and policymakers similarly highlighted that “*difficulties reading text,*” “*visual impairments*” [PV004], “*hands and feet less nimble*” [PB002], and “*cognitive issues*” [SH001] could hinder independent use of the app. This suggested that functional and health-related limitations could further reduce the accessibility of HA Go.

#### Healthcare providers’ familiarity with HA Go

Physicians and policymakers also suggested that limited provider familiarity may reduce the practical integration and impact of HA Go in clinical settings (Table 5). They emphasized that healthcare professionals needed to be more “*familiar with the app*” [PB008], and that limited awareness of “*new functions*” could reduce their ability to promote or explain HA Go to patients [SH006].

### Individuals—motivation

#### Perspectives on privacy protection

Participants across stakeholder groups expressed mixed perspectives on privacy protection in relation to HA Go (Tables 4 and 5). On the one hand, some users considered HA Go and similar online applications to be safe and felt “*very at ease*” using them [UP003]. On the other hand, both end-users and healthcare stakeholders raised concerns about privacy risks, including the need to verify identity documents, the possibility of “*data leak[s]*” [NU002], and the risk that personal information could be exposed if a phone was lost [PB004]. These findings highlighted privacy and data security as important considerations in decisions about adopting HA Go.

#### Motivation and limitations to use HA Go

Physicians and policymakers noted that motivation to use HA Go depended partly on whether patients were willing to take a more active role in managing their own health information (Table 5). One policymaker referred to patients wanting to “*take control of*” their “*own information*” and “*own medical condition*” [SH001], whereas one physician noted that adoption could be limited when patients were unwilling to accept “*this new approach*” [PB002]. This suggested that willingness to adopt HA Go was shaped not only by its functions, but also by readiness to change existing habits.

#### Personal preference

However, willingness to adopt HA Go did not always translate into actual use, as personal preference could still influence how participants chose to seek care (Table 4). One non-user noted that when feeling unwell, they would rather “*go to see a private doctor*” than use HA Go to book an appointment [NU001], which suggested that adoption might remain limited when HA Go does not align with users’ usual care-seeking preferences.

#### Worries about operation errors

Motivation to use HA Go could also be undermined by concerns about operation errors. Physicians noted that some older adults were reluctant to use the app for tasks such as payment or appointment booking because they feared making “*errors*” in “*scanning*” or “*booking*” [PV001].

#### Limited motivation due to unfamiliarity with digital devices and interface

Related to these concerns about operation errors, physicians and policymakers also noted that unfamiliarity with the interface could weaken willingness to continue using HA Go (Table 5). One physician observed that some older adults might give up using the app if they “*cannot find what they want after pressing a few buttons*” [PB004].

#### Low motivation to learn and use

This reduced motivation could be reinforced when users were unwilling to invest time in learning how to use HA Go (Table 4). Some users described resistance to “*learn new things*” [UP002] and felt that learning to use such apps would “*waste a lot of time*” [UP005].

### Individuals—opportunity

#### External influences on patient decisions

Interpersonal support was perceived as an important facilitator of HA Go adoption among users, non-users, and caregivers (Table 4). Participants referred to external influence from “*some friends who use it*” [UP005] and from “*family members and some healthcare personnel*” who “*require[d] me to use it*” [NU004], suggesting that interpersonal encouragement and support could promote the uptake of HA Go.

#### Influence and assistance from others

A similar role of interpersonal support was also noted by physicians and policymakers (Table 5). One physician noted that patients’ use of HA Go could be influenced by “*family members or peers*” and often spread through “*word of mouth*” [PB004].

#### Availability of time to explore HA Go

The availability of time to explore HA Go had mixed effects on its use among users and non-users (Table 4). One user suggested that they could “*take a look*” at the app when there was time [UP001], whereas another non-user noted that caregiving responsibilities meant they “*don’t have the time*” to engage with it [NU005].

#### Availability of time to use in private and public healthcare settings

A similar concern about time was also raised by physicians and policymakers, who noted that limited time in both private and public healthcare settings could restrict the practical use of HA Go (Table 5). One physician pointed out that the clinical environment involved “*time constraints,*” which left less opportunity to use the app meaningfully during practice [PV004]. This suggested that beyond users’ own time to explore the app, time pressures in healthcare settings could also limit its routine use.

#### Device and internet issues

Practical problems with devices and internet access were also identified as barriers to the use of HA Go among users, non-users, and caregivers, especially when app access depended on a functioning personal device, sufficient battery, and stable internet connection (Table 4). One user noted that using HA Go could “*waste my data*” when Wi-Fi access was inadequate [UP005].

## Outer setting

### Outer setting—local conditions

#### Inevitability of digitalization

Physicians and policymakers viewed HA Go as aligned with the wider trend toward digitalization in healthcare and public services (Table 5). One physician described this as an “*inevitable trend*” toward a more “*paperless*” system and considered it “*definitely necessary*” for a large healthcare organization such as HA [PB002].

## Inner setting

### Inner setting—access to knowledge and information

#### Availability of knowledge resources for patients and healthcare professionals

Participants across stakeholder groups expressed mixed views regarding the availability of knowledge resources related to HA Go (Tables 4 and 5). While users, non-users, and caregivers generally perceived information as accessible, some noted limitations in the quality of support, particularly from volunteers, who “*wouldn’t know how to respond*” to further questions [UP005]. Policymakers highlighted the availability of official guidance, such as “*step-by-step instructions*” on dedicated webpages [SH004]. However, physicians raised concerns about limited access to HA Go-related information for both patients and healthcare professionals, which they felt hindered adoption in clinical settings. One physician considered current patient awareness “*not enough*” [PV002], while another noted that staff were expected to understand the app “*just from an email*” [PB008]. Overall, these findings indicated that although HA Go-related information was available, gaps in dissemination and support may still hinder its effective use and adoption.

#### Training and education required

The perceived gap in practical support was further reflected in calls from users, non-users, and caregivers for training and education to support HA Go adoption (Table 4). One user suggested offering classes for those who “*don’t know how to use it*” or are “*afraid to use it*” [UP002], indicating a need for more structured user support.

### Inner setting—compatibility

#### Compatibility with existing medical system

Physicians and policymakers reported differing views on how well HA Go fit within the existing medical system (Table 5). Some considered the platform compatible with HA’s service structure and broader digitalization efforts, describing it as “*a good support system*” for public healthcare services [PB002]. Others, however, noted that HA Go was still “*not fully integrated*” with existing systems such as the clinical management system (CMS) [SH006], which may limit its practical value in routine healthcare delivery. These findings suggested that system compatibility was an important organizational factor shaping the implementation of HA Go.

#### Limited impact on actual workflow

Despite this perceived compatibility, some physicians felt that HA Go had limited impact on their actual clinical workflow (Table 5). As one physician noted, “*I can access eHealth from my own hospital, I don’t see what additional information HA Go can provide me*” [PV002].

### Inner setting—available resources

#### Lack of resources

Beyond concerns about workflow relevance, physicians also highlighted a lack of resources as a barrier to implementation (Table 5). One physician noted that “*we really need additional clinical staff*” to promote HA Go and teach patients how to use it [PB006], suggesting that limited manpower may constrain wider uptake in practice.

### Inner setting—incentive systems

#### Rewards encouraging use

Compared with broader organizational barriers, some participants viewed small incentives as a modest facilitator of uptake. One user reported that receiving a small gift made the app more “*appealing*” [UP005], indicating that small rewards may support early uptake (Table 4).

## Implementation process

### Implementation process—engaging

#### Promotion for patients needed

Users and caregivers indicated that more visible patient-facing promotion was needed to support the adoption of HA Go (Table 4). One participant suggested that there should be “*publishing notices*” or “*individuals promoting it*” in places such as clinics [UP002], while another felt that there was little visible promotion “*on TV, or on the radio, in newspapers*” [CG001]. These findings suggested that stronger and more visible outreach might help improve public awareness and uptake of HA Go.

#### Improvement in publicity strategies

Physicians and policymakers also highlighted diversified publicity strategies that may enhance patient engagement in HA Go adoption and usage, including feature-based advertising and community outreach (Table 5). For example, a physician indicated, *“I think there should be a bit more advertising or campaigns to inform patients about what HA Go can do ”*[PV001].

#### Insufficient promotion of HA Go’s existence and functions for patients and physicians

One physician pointed out that promotion and accessibility support for HA Go were “*not enough*” [PV002], suggesting that limited awareness of the app’s existence and functions among both patients and healthcare professionals could hinder its uptake (Table 5).

### Implementation process—teaming

#### Cross-department collaboration required for advocacy

Physicians and policymakers emphasized that broader promotion of HA Go would also require cross-department collaboration (Table 5). One policymaker noted that this would need “*discussions with some primary care stakeholders*” [SH002], suggesting that coordination across different sectors and service levels could support more effective advocacy and implementation of HA Go.

### Implementation process—assessing context

#### Perceived barriers and risks for implementation

Physicians and policymakers also highlighted potential risks that could complicate the implementation of HA Go (Table 5). One policymaker raised concern that caregiver-related functions might expose older adults to being “*scammed*” [SH001], indicating that implementation was shaped not only by promotion and coordination, but also by concerns about safety, monitoring, and operational capacity.

### Implementation process—reflecting and evaluating

#### Need for feedback loops

Given these implementation challenges, physicians and policymakers highlighted the need for feedback loops to improve HA Go over time (Table 5). A physician remarked that some previously reported suggestions had remained “*frozen*” [PB003], pointing to the need for a more responsive mechanism for ongoing improvement.

### Implementation process—adapting

#### Timely updates and improvements needed

Related to the need for ongoing feedback and refinement, one caregiver emphasized that HA Go should “*keep developing*” and become “*more convenient*” over time [CG001] (Table 4). This highlighted that timely updates may help sustain users’ interest in the app.

## Discussion

### Summary of findings

This qualitative study examined the perspectives of 33 key stakeholders on the adoption of HA Go in Hong Kong and provided a comprehensive understanding of shared and divergent views across users, non-users, caregivers, physicians, and policymakers. Across all groups, participants consistently recognized the significance of HA Go in appointment management and the streamlining of healthcare processes, which were widely cited as the key advantages that facilitate its adoption. However, they expressed concern that patients’ lack of familiarity with electronic devices might hinder the wider adoption of HA Go, particularly among older adults.

Among user, non-user, and caregiver groups, most participants highlighted that easy access to HA Go-related information further supported adoption. In contrast, physicians and policymakers expressed more mixed views. While some policymakers highlighted the availability of official support resources, such as dedicated webpages, many physicians noted that current access to information was insufficient for both patients and healthcare professionals, limiting effective adoption in clinical settings. Furthermore, physicians and policymakers offered broader perspectives on HA Go’s benefits, emphasizing improvements in medication tracking, health management, quality of caregiving services, physician-patient communication, and optimization of healthcare resources. Suggested improvements to HA Go were consistent across groups and primarily focused on enhancing existing functionality and optimizing the user interface to improve future viability. In addition, physicians and policymakers advocated for the integration of existing platforms and the reduction of functional duplication to alleviate the burden on the public, as well as improvements in promotion and outreach strategy to enhance patient engagement.

### Comparison with previous literature

HA Go is a mobile application that integrates appointment management, payment, medication acquisition, and health management, thereby addressing diverse users’ needs for digital health services. Our findings underscored the benefits of using HA Go in enhancing healthcare delivery. Users can easily book Family Medicine Clinic (FMC) appointments through HA Go’s “Book FMC” feature, which is user-friendly and accessible.^30^ The option to schedule appointments for family members is particularly beneficial for older adults who require support in managing healthcare arrangements. Additionally, the app allows users to share appointment details via email through the “Share” option, simplifying communication and reducing the risk of missed appointments.^30^ These functions were consistently recognized by stakeholders as facilitators of adoption, especially among older adults, through simplified processes and greater autonomy.

Health self-management emerged as a key benefit of HA Go. Consistent with our findings, a qualitative study on the use of health apps for self-health care and self-monitoring showed that the use of health apps can bring many perceived benefits.^31^ A study in China also found that mobile health applications could improve healthcare convenience by enabling post-consultation payment and reducing queuing time, while also enhancing users’ care experience, particularly in communication and access to health information.^32^ Mobile health applications may also improve patients’ experience with payment and medical services fee information, thereby supporting greater transparency in healthcare service charges.^33^ Overall, mobile health applications are believed to improve service efficiency in the medical service sector.^34^

A notable divergence emerged between stakeholder groups regarding access to HA Go-related information. While users and caregivers generally felt that information was accessible, physicians and policymakers expressed concerns about insufficient communication and training. Physicians noted that reliance on email updates was ineffective for many healthcare staff, limiting their ability to support patients. This discrepancy may reflect differences in expectations and roles: end-users may focus on surface-level usability, while healthcare professionals require more structured training and clearer integration of HA Go into clinical workflows. Similar concerns have been raised in other studies, such as a qualitative study of French general practitioners, highlighted the need for appropriate education and training, improved digital skills, and better interoperability, while also noting perceived risks such as patient isolation, privacy and data security concerns, additional workload, and over-medicalization associated with mobile health technologies.^35^

Our study also highlighted concerns about patients’ familiarity with electronic devices, particularly among older adults. This barrier is well-documented in the literature, with studies showing that older users often struggle with learning and using new technologies.^36^^,^^37^ Factors such as ease of use and social influence—encouragement from family or healthcare providers—are important for adoption. Previous research suggests that effort expectancy and peer support are associated with mHealth acceptance, supporting the need for user-centered design and community-based promotional strategies.^36^^,^^37^ Community support and training for older adults are also necessary since mobile health applications have shown promise in leveraging community resources to support patients with chronic conditions, including older adults and other underserved patient groups.^38–40^ Our findings align with this, suggesting that initial training and ongoing support are essential for older users. Such interventions have been shown to enhance digital literacy and increase confidence in using digital technologies among older populations.^41^^,^^42^

Given the barriers identified, participants emphasized the need to enhance HA Go’s future viability by refining its user interface, expanding functionality, and integrating it with existing systems to reduce redundancy. These recommendations are consistent with calls in the literature for standardized evaluation frameworks to assess the effectiveness and safety of mobile health applications.^43^^,^^44^ Accountability has been identified as an important factor in effective app implementation, covering not only the medical effects achieved through the apps but also their potential unintended consequences and associated changes in healthcare utilization patterns.^45^ Moreover, concerns about app reliability and trustworthiness have been echoed in a previous study, which suggested that users’ intention to adopt mHealth services may be influenced by factors such as system liability, privacy, trust, effort expectancy, and performance expectancy.^46^ To address this, mobile health applications should be grounded in credible scientific evidence and subject to regular review to ensure user confidence and sustained engagement.^47^

### Strengths and limitations

A key strength of this study lies in its adoption of qualitative methods, particularly in-depth interviews with a diverse range of stakeholders. This approach captured nuanced perspectives on HA Go adoption, revealing critical barriers and facilitators that might have been overlooked in quantitative assessments. Additionally, our research achieved comprehensive representation across all key user groups, including user, non-user, and caregiver groups, physician groups actively involved in HA Go implementation, and policymaker groups responsible for eHealth strategy. This multi-stakeholder approach enabled the study to capture perspectives from both frontline users and decision-makers.

However, this study has several limitations. First, the adoption of purposive sampling may have introduced selection bias. Notably, all interviewed users, non-users, and caregivers were aged ≥60 years, predominantly female, with low household incomes and high chronic disease prevalence. Consequently, perspectives from younger, male, or higher-income populations were underrepresented, potentially limiting the generalizability of our findings. Second, the findings are subject to strong temporal limitations, as stakeholder perspectives on HA Go adoption may evolve over time due to app updates (eg, feature enhancements), policy reforms, or shifting user experiences. As this study captured perceptions at a single time point, findings might not reflect long-term attitudes or post-intervention changes. Third, although the qualitative data revealed positive stakeholder views, these attitudes do not necessarily translate into actual adoption rates or sustained engagement. The absence of quantitative metrics (eg, app usage statistics, dropout rates) limits the ability to assess the real-world impact of identified facilitators or barriers on actual adoption.

### Implications

The identification of facilitators and barriers to HA Go adoption has significant implications for intervention development and policy reform. Given concerns about older users’ familiarity with electronic devices, healthcare providers could implement training programs, such as in-person tutorials, to bridge the digital divide and improve the utilization of HA Go. Furthermore, our study found that while patients appreciated the accessibility of HA Go, physicians and policymakers expressed concerns about the depth of available information. A two-tiered information system that provides basic information for patients and detailed information for professionals may address these differing needs and support wider adoption.

## Conclusions

This qualitative study explored HA Go adoption in Hong Kong, revealing both shared and divergent views across users, non-users, caregivers, physicians, and policymakers. While all stakeholder groups acknowledged HA Go’s role in streamlining appointment management and healthcare processes, concerns about digital literacy—especially among older adults—emerged as a consistent barrier to adoption. Differences in perceived access to HA Go–related information highlighted gaps in support for clinical integration. These insights point to the need for targeted improvements in user interface design, platform integration, and communication strategies to foster wider adoption and sustain long-term engagement.

## Supplementary Material

ooag133_Supplementary_Data

## Data Availability

The datasets used and/or analyzed during the current study are available from the corresponding author on reasonable request.
